# Diagnostic Value of Increased [18F]FDG Uptake in Locoregional Lymph Nodes on PET/CT in Patients with Suspected Fracture-Related Infection

**DOI:** 10.3390/diagnostics15050616

**Published:** 2025-03-04

**Authors:** Paul Bosch, Andor W. J. M. Glaudemans, Jean-Paul P. M. de Vries, Johannes H. van Snick, Justin V. C. Lemans, Janna van den Kieboom, Monique G. G. Hobbelink, Geertje A. M. Govaert, Frank F. A. IJpma

**Affiliations:** 1 Department of Surgery, University Medical Center Groningen, University of Groningen, 9712 CP Groningen, The Netherlandsj.p.p.m.de.vries@umcg.nl (J.-P.P.M.d.V.); f.f.a.ijpma@umcg.nl (F.F.A.I.); 2 Department of Nuclear Medicine and Molecular Imaging, University Medical Center Groningen, University of Groningen, 9712 CP Groningen, The Netherlands; j.h.van.snick@umcg.nl; 3 Department of Trauma Surgery, University Medical Center Utrecht, University of Utrecht, 3584 CS Utrecht, The Netherlands; j.v.c.lemans-3@umcutrecht.nl (J.V.C.L.); g.a.m.govaert@umcutrecht.nl (G.A.M.G.); 4 Department of Radiology and Nuclear Medicine, University Medical Center Utrecht, University of Utrecht, 3584 CS Utrecht, The Netherlands

**Keywords:** [18F]FDG-PET/CT, fracture-related infection, FRI, lymph nodes, infection, imaging

## Abstract

**Background:** Diagnosing fracture-related infection (FRI) without clinical confirmatory signs is challenging. [18F]FDG-PET/CT has been shown to have good diagnostic accuracy. However, direct interpretation criteria are lacking. The aim of this study was to assess the diagnostic value of increased FDG-uptake in locoregional lymph nodes on [18F]FDG-PET/CT in patients with suspected upper and lower extremity FRI. **Methods**: This was a retrospective cohort study of patients who underwent [18F]FDG-PET/CT for suspected extremity FRI in two tertiary referral centers between January 2011 and December 2023. The sensitivity, specificity and diagnostic value of the presence, number and intensity of [18F]FDG uptake in locoregional lymph nodes was assessed. Uptake intensity was measured by calculating the maximum standard uptake value (SUVmax) of the ‘hottest’ lymph node. All scans were acquired according to the European Association of Nuclear Medicine (EANM) standards, and quantification was performed based on standardized EARL reconstructed images. FRI was diagnosed based on positive intra-operative microbiology results or development of clinical confirmatory signs within six months of follow-up. **Results**: One-hundred-and-twenty-four patients were included in the analysis, with 71 cases of confirmed FRI. The presence of locoregional lymph nodes alone showed poor diagnostic accuracy (sensitivity 55%, specificity 68%, diagnostic accuracy 62%). The number of active lymph nodes showed poor discriminative performance between FRI and non-infectious cases (AUC 0.63). Utilizing the SUVmax of the ‘hottest’ lymph nodes showed a moderate discriminative performance with an AUC of 0.71. The optimal cutoff point (SUVmax 3.48) resulted in a sensitivity of 72%, a specificity of 78% and a diagnostic accuracy of 75%. A logistic regression model was fitted to calculate the added value of lymph node assessment to the regular [18F]FDG-PET/CT assessment. This resulted in a sensitivity of 71%, a specificity of 82% and a diagnostic accuracy of 76%. **Conclusions**: Presence and number of locoregional lymph nodes with increased [18F]FDG-uptake alone has poor diagnostic accuracy for FRI. The SUVmax of the ‘hottest’ lymph node showed moderate diagnostic performance. Lymph node assessment slightly increased the diagnostic value of regular [18F]FDG-PET/CT assessment. Based on these results, increased [18F]FDG-uptake in locoregional lymph nodes should only be considered as a suggestive sign for a positive scan result in suspected FRI.

## 1. Introduction

Fracture-related infection (FRI) is a severe complication following surgical fracture treatment. The mechanisms involved are complex and can significantly impact patient morbidity and healthcare costs [1,2,3,4]. Early and accurate detection of FRI is essential for optimizing patient outcomes [5]. This can be challenging in patients presenting with only suggestive clinical signs of FRI. A recent study showed that almost a quarter of patients with suspected FRI do not show clinical confirmatory signs at first presentation [6]. In these cases, additional diagnostic tests are often performed, including laboratory studies and radiological and/or nuclear imaging. According to the 2018 Arbeitsgemeinschaft für Osteosynthesefragen (AO) and European Bone and Joint Infection Society (EBJIS) FRI consensus definition, signs of infection on nuclear imaging are considered as being suggestive for the presence of FRI [7]. White Blood Cell (WBC) scintigraphy and [18F]FDG-PET/CT have been shown to have the highest diagnostic accuracy for FRI, and are routinely used in clinical practice [8,9,10]. However, definitive image acquisition and interpretation guidelines based on prospective data are still lacking [11]. Optimizing the diagnostic performance of nuclear imaging techniques is essential to prevent false positive and false negative FRI diagnoses in cases with absent confirmatory criteria, thereby preventing over- and undertreatment.

[18F]FDG-PET/CT has the ability to detect increased glucose metabolism irrespective of its cause. Therefore, increased metabolism can be present in both infectious and non-infectious causes, such as high glucose metabolism due to increased osteoblast activity at fracture sites or inflammation due to (surgical) trauma or as a reaction on foreign body material. Therefore, distinguishing infection from sterile inflammation in patients with suspected FRI can be challenging [12]. Quantifying findings on [18F]FDG-PET can be a useful tool in differentiating between inflammation and infection, such as calculating the Standardized Uptake Value (SUV) [13]. A previous study by our group on the diagnostic accuracy of [18F]FDG-PET/CT for FRI showed that SUVmax calculations of [18F]FDG-uptake at the FRI region of interest increased the diagnostic performance when compared to visual (qualitative) analysis alone [8].

Besides quantification tools at the FRI region of interest, assessment of possible secondary signs of infection can also be used to maximize the diagnostic performance of [18F]FDG-PET/CT. Locoregional lymphadenopathy with increased [18F]FDG-uptake has been studied as a secondary sign of infection in other fields, such as in infectious endocarditis and vascular graft infections [14,15]. In [18F]FDG-PET/CT scans of suspected upper- and lower extremity FRI, locoregional axillary or inguinal/iliac lymph nodes are often included in the field-of-view, and may therefore provide additional diagnostic information. Therefore, the aim of this study was to assess the diagnostic value of increased [18F]FDG-uptake in locoregional lymph nodes on [18F]FDG-PET/CT in patients with suspected extremity FRI.

## 2. Materials and Methods

### 2.1. Study Design and Population

A retrospective cohort study was performed. Patients who underwent [18F]FDG-PET/CT for suspected upper and lower extremity FRI at two tertiary referral centers [University Medical Center Groningen (UMCG), Groningen, The Netherlands, and University Medical Center Utrecht (UMCU), Utrecht, The Netherlands] between January 2011 and December 2023 were eligible for inclusion in this study.

### 2.2. Exclusion Criteria

1: Locoregional (axillary or inguinal/iliac lymph nodes) not included in the scan field-of-view.

2: No available reference standard for FRI as defined in the 2018 AO/EBJIS consensus definition.

3: Non-extremity FRI (e.g., pelvis, spine, etc.).

4: Presence of a comorbidity associated with locoregional lymphadenopathy (e.g., malignancy, auto-immune disorders, other infectious focus in affected limb).

### 2.3. Patient Information

Information regarding patient gender, age, AO and Gustilo–Anderson classification of initial fracture, relevant comorbidity, medical microbiology results and clinical follow-up was retrospectively collected. Dates of initial fracture and the last surgery preceding the [18F)FDG-PET/CT were collected.

### 2.4. FDG-PET/CT Acquisition and Assessment

Patient preparation and scan acquisition for the [18F]FDG-PET/CT scan were according to EANM guidelines [16]. In short, patients had a minimum of a 6 h fasting period before the administration of 2–3 MBq/kg [18F]FDG. Scan acquisition was performed 60 +/− 5 min after the injection, with an acquisition time of 3 min per bed position. All quantification measurements were performed on EANM Research Ltd. (EARL) standardized reconstructions [17]. All [18F]FDG-PET/CT scans were evaluated visually and quantitatively via the Syngo.via VB50-60 software system [Siemens Healthcare GmbH ©, Knoxville, TN, USA]. All scans were assessed for the presence of FRI based on the following signs: uptake location and pattern, intensity of uptake by comparing the region of interest with the contralateral side, and involvement of bone, muscle and osteosynthesis material. Then, scans were visually assessed (PB) for the presence of locoregional (axillary in case of suspected upper extremity FRI, or inguinal/iliac in case of suspected lower extremity FRI) lymph nodes with increased [18F]FDG-uptake. If there were lymph nodes with increased [18F]FDG-uptake, the number of active lymph nodes was assessed and the SUVmax and SUVmean were calculated.

### 2.5. Reference Test

In case of operative management, the diagnosis was based on at least two surgically obtained deep tissue cultures, with at least two separate samples showing the same pathogen, *or* the presence of clinical confirmatory signs (e.g., fistula/sinus/pus) during the operation. Both are considered the gold standard as defined in the 2018 AO/EBJIS consensus definition on FRI [7]. In case of non-operative management: at least twelve months clinical follow-up by a trauma surgeon was performed. During this period, the presence of clinical confirmatory signs (e.g., fistula/sinus/pus) would indicate a positive diagnosis of FRI.

### 2.6. Statistical Analysis

True positive (TP), true negative (TN), false positive (FP) and false negative (FN) results were assessed and displayed in contingency tables. Sensitivity, specificity and diagnostic accuracy were calculated. Analysis was performed for both qualitative (presence or absence of lymphadenopathy) and quantitative assessment (SUV-max of ‘hottest’ lymph node present). Secondly, the highest SUVmax per patient was used as continuous values to calculate the area under the receiver operating characteristic (AUROC) as a measure of discriminative performance. Lastly, because the assessment of lymph nodes is always performed in the context of assessment of [18F]FDG-uptake in the region of suspected FRI, a binary logistic regression model was fitted to include both the evaluation of the suspected FRI focus and the evaluation of locoregional lymph nodes, and compared to the diagnostic performance of the regular scan assessment, to test if the presence of increased [18F]FDG-uptake in locoregional lymph nodes increases the diagnostic yield of the scan. All data analyses were performed using the Statistical Package for Social Sciences (SPSS^®^) statistics for Windows (version 20.0.0.0, IBM, Armonk, NY, USA).

## 3. Results

### 3.1. Population Characteristics

**Figure 1:**
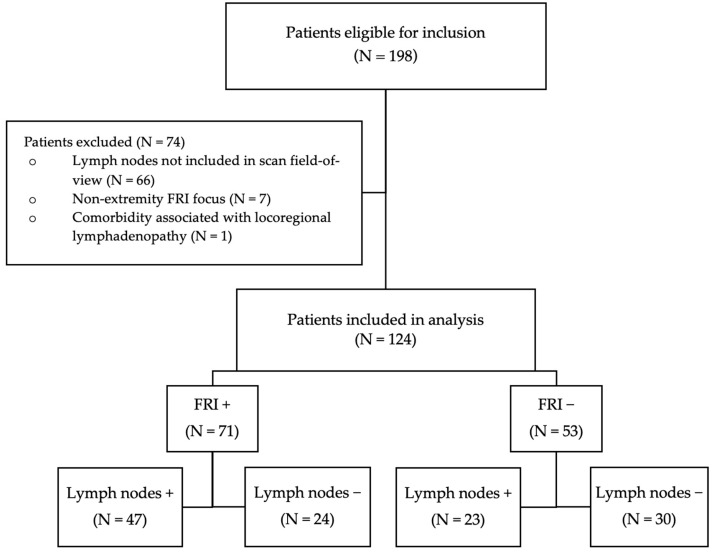
Flowchart of patient inclusion.

After exclusion, a total of 124 patients were included in the analysis. Patient inclusion is visualized in Figure 1. Patient characteristics are displayed in Table 1.

### 3.2. FRI in Study Population

Of 124 included patients, 71 (57%) were diagnosed with FRI based on the AO/EBJIS consensus definition. Of the 71 FRI cases, 55 (77%) were diagnosed based on intra-operative deep tissue cultures, and 16 were diagnosed based on clinical confirmatory signs during a follow-up of at least 6 months after the first clinical suspicion of FRI.

### 3.3. Diagnostic Accuracy of Standard Assessment Protocol

First, the diagnostic performance of the regular [18F]FDG-PET/CT assessment was calculated using crosstabs, yielding 51 true positive results, 16 false negative results, 41 true negative results and 17 false positive results. This resulted in a sensitivity of 76% (62–87%), a specificity of 71% (58–81%), and a diagnostic accuracy of 73% (64–81%).

### 3.4. Diagnostic Accuracy of Presence and Number of FDG-Positive Lymph Nodes

Of 124 patients included, 52 (42%) showed increased [18F]FDG-uptake in axillary, inguinal or iliac lymph nodes on [18F]FDG-PET/CT. Of these patients, 29 (56%) were diagnosed with FRI according to the reference standard. The diagnostic performance based on the qualitative assessment (e.g., presence or absence of increased [18F]FDG-uptake in locoregional lymph nodes) yielded 29 true positive results, 24 false negative results, 48 true negative results and 23 false positive results. This resulted in a sensitivity of 55% (40.45–68.44%) and a specificity of 68% (55.45–78.24%), with a diagnostic accuracy of 62% (52.95–70.65%).

**Figure 2:**
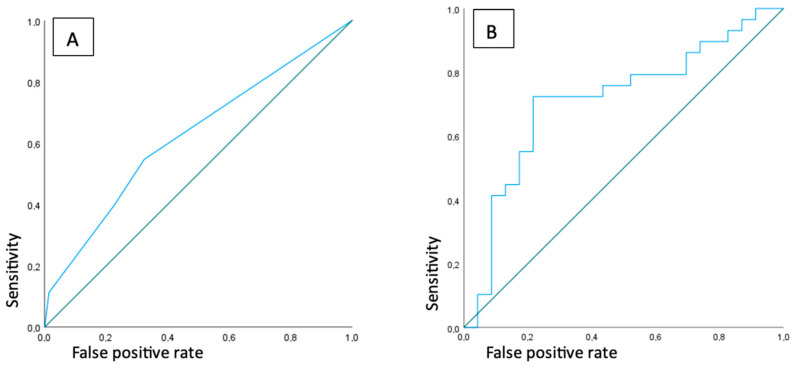
ROC curves representing discriminative ability of presence of lymph nodes with increased uptake (**A**) and highest SUVmax of locoregional lymph nodes (**B**) for FRI.

**Figure 3:**
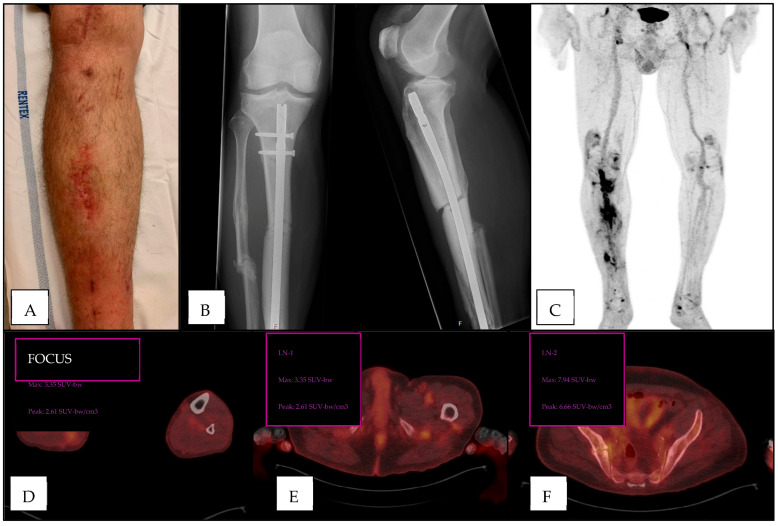
Patient presenting with local erythema and ulceration but no clinical confirmatory signs for FRI, three months after crush injury of the right lower leg with open fracture of the tibia and fibula (**A**). Plain radiograph with intramedullary nail in situ (**B**). [18F]FDG-PET/CT showed increased uptake at the fracture site suggestive for infection (**C**,**D**). Inguinal (**E**) and iliac (**F**) lymph nodes with increased uptake were seen. Intra-operatively obtained deep tissue cultures were positive for *Staphylococcus aureus*.

**Figure 4:**
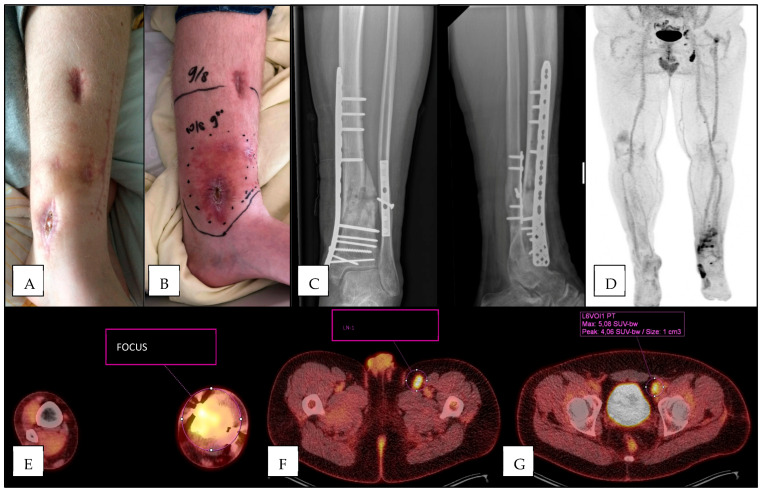
Patient presenting 17 months after distal tibia fracture, and three months after non-union treatment with autologous cancellous bone graft. Initially, there were no clinical confirmatory signs (**A**), though progressive pain and erythema developed in the following weeks (**B**) suggestive for FRI. Plain radiograph showed non-union after plate- and screw osteosynthesis (**C**). [18F]FDG-PET/CT was suggestive for FRI with increased uptake at the fracture site (**D**,**E**), and showed inguinal (**F**) and iliac (**G**) lymph nodes with increased uptake. Intra-operatively obtained deep tissue cultures were positive for *Staphylococcus epidermidis*.

**Figure 5:**
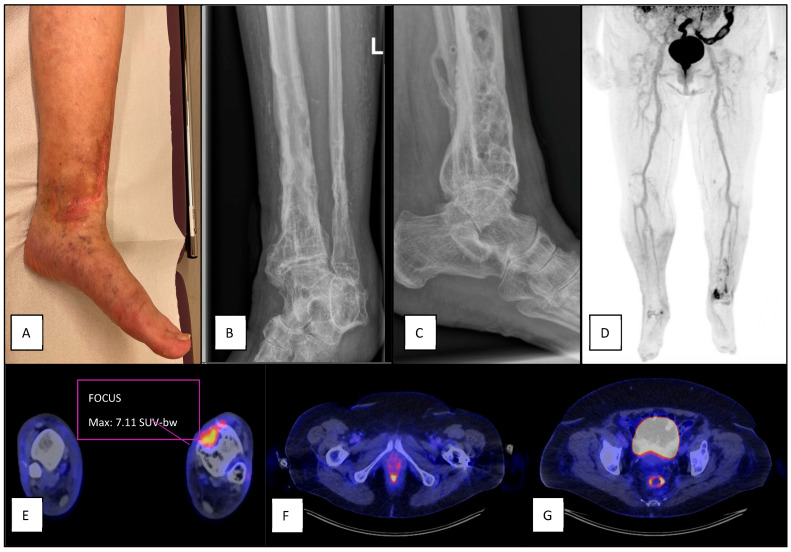
Patient presented 18 years after sustaining a distal tibia fracture for which a plate and screw osteosynthesis was performed. The plate and scews were later removed. The patient presented with local pain and erythema (**A**), but no clinical confirmatory signs. Plain radiograph showed extensive post-traumatic bone deformity (**B**,**C**). FDG-PET/CT showed increased FDG-uptake at the distal tibia (**D**,**E**). No increased FDG-uptake was seen in inguinal (**F**) or iliac (**G**) lymph nodes. Intra-operative cultures were positive for *Corynebacterium propinquum*.

Of the 52 patients with positive lymph nodes, 15 patients had a single positive lymph node, 22 had two positive lymph nodes, 8 patients had three positive lymph nodes, and 7 patients had four or more positive lymph nodes. The AUROC was calculated using the number of lymph nodes with increased [18F]FDG-uptake as a measure of discriminative performance for the presence of FRI, which resulted in an AUROC of 0.63, indicating a poor discriminative performance (Figure 2A). Three cases are exemplified in Figure 3, Figure 4 and Figure 5.

### 3.5. Highest SUVmax

For the 52 patients with increased [18F]FDG-uptake in locoregional lymph nodes, the highest SUVmax per patient was used to calculate the area under the receiver-operated characteristic. This resulted in an AUROC of 0.71 (0.56–0.86), indicating a modest discriminative performance for FRI (Figure 2B). The best diagnostic performance was seen with a SUVmax cutoff point of 3.48, which resulted in a sensitivity of 72% (52–87%), a specificity of 78% (56–93%) and a diagnostic accuracy of 75% (61–86%).

### 3.6. Added Value of Locoregional Lymph Node Assessment

To test if adding lymph node assessment to the regular assessment of the [18F]FDG-PET/CT in FRI would increase diagnostic performance, a logistic regression analysis was performed. The model was fitted with both the regular [18F]FDG-PET/CT assessment model (e.g., FRI yes/no, based on the criteria listed in Section 2.4), and the presence of lymph nodes with a SUVmax > 3.48 (yes/no) model as predictors of FRI. This model was then compared to the gold standard for FRI as defined in Section 2.5. This resulted in a sensitivity of 71% (51–87%), a specificity of 82% (60–95%) and a diagnostic accuracy of 76% (62–87%).

## 4. Discussion

This study investigated whether the presence of increased [18F]FDG-uptake in locoregional lymph nodes was of added value for diagnosing FRI in a large patient cohort and with standardized image acquisition and quantification. The presence of increased [18F]FDG-uptake in locoregional lymph nodes alone was not predictive for FRI, and a larger number of [18F]FDG-PET positive lymph nodes was not discriminative for FRI. The intensity of [18F]FDG-uptake (by calculation of the SUVmax) in locoregional lymph nodes showed a moderate diagnostic value, with an optimal SUV-max cutoff point of 3.48. The addition of lymph node assessment to the standard interpretation protocol of [18F]FDG-PET/CT slightly increases the diagnostic performance when compared to the standard assessment alone.

Only one other study reported on the diagnostic value of locoregional lymph nodes in cases of suspected FRI [18]. Wang et al. reported a good diagnostic accuracy for the SUVmax of inguinal lymph nodes in cases with suspected FRI. Their retrospective cohort study included 254 patients, with a definite diagnosis of FRI in 197 patients. The AO/EBJIS consensus definition was used as a reference standard for diagnosing FRI, though no information regarding follow-up was specified. They found a sensitivity of 86.8%, a specificity of 93.0% and an AUC of 0.939 for the SUVmax measurement in the ‘hottest’ inguinal lymph node. Furthermore, the diagnostic accuracy using the SUVmax in inguinal lymph nodes was higher than the diagnostic accuracy using the SUVmax at suspected FRI sites. Unfortunately, they did not report the number of patients with confirmed FRI that showed increased [18F]FDG-uptake in locoregional lymph nodes. Furthermore, scan acquisition and quantitative analysis were not standardized. In our cohort, only 66% of patients who were diagnosed with FRI showed increased FDG-uptake in locoregional lymph nodes.

Studies evaluating the added value of metabolically active lymph nodes in other infectious diseases are available. Van Rijsewijk et al. reported on the added value of locoregional lymphadenopathy in suspected abdominal vascular graft and endograft infections (VGEI) [14]. They found a high specificity of 96%, with none of the non-infectious cases having locoregional lymph nodes with increased [18F]FDG-uptake. However, the number of false negative scans was fairly high, resulting in a poor sensitivity of 35%. Their results may have been influenced by the very small group of non-infected cases (6.6%). Ten Hove et al. investigated the added diagnostic value of mediastinal lymphadenopathy on [18F]FDG-PET/CT in the context of suspected infectious endocarditis [15]. They found no added value of increased [18F]FDG-uptake in locoregional lymph nodes, also reporting a high number of false positive results.

The results of the current study may have significant clinical relevance, since they suggest that the addition of lymph node assessment to the standard interpretation of [18F]FDG-PET/CT in suspected FRI marginally increases its diagnostic yield. Increasing the diagnostic yield of non-invasive diagnostic tests is essential in cases of suspected FRI where clinical confirmatory symptoms are absent. The current study and recent literature underline the essential place that nuclear imaging has in the diagnostic workup of clinically non-evident FRI. However, the current evidence in the literature is still sparse, and consists solely of retrospective data. Future perspectives for increasing the diagnostic yield of [18F]FDG-PET/CT for FRI may come from developments in infection/inflammation-specific tracers, pathogen-specific tracers, hybrid imaging, variations in scanning protocols, such as dual time point imaging, and recent developments in large-axial-field-of-view camera systems with higher sensitivity [19]. Diagnosing fracture-related infection (FRI) is challenging because bacterial infections are difficult to distinguish from sterile inflammation caused by factors such as soft tissue injury, fractured bone, surgery, and implanted osteosynthesis devices. Additionally, during revision surgery for suspected FRI, differentiating between infected and healthy tissue remains complex. Bacteria-targeted molecular imaging, particularly fluorescence imaging and PET/CT, offers a promising solution to improve diagnostic accuracy [20,21,22]. Fluorescence imaging detects bacterial infections in real time using a fluorophore-coupled target-specific agent and an optical camera. Recent studies demonstrated that vancomycin-IRDye800CW, a conjugate of vancomycin and IRDye800CW, is effective for imaging FRI by targeting Gram-positive bacteria [20]. Recently, our group developed bacteria-targeted PET tracers, including [18F]PQ-VE1-vancomycin [21,22]. These novel tracers aim to enhance infection imaging and diagnostic accuracy. We believe that novel bacteria-targeted PET tracers enable preoperative noninvasive detection and quantification of fracture-related bacterial infections, while fluorescence imaging offers real-time intraoperative imaging of bacterial biofilms before and after surgical debridement. Future research will focus on translating these preclinical findings into clinical applications.

This study has some strengths and some limitations. The current study has, for the first time, assessed the value of locoregional lymph node assessment to the standard [18F]FDG-PET/CT interpretation in cases of suspected extremity FRI, performed in a standardized setting, with scan acquisition, interpretation, and quantification based on European standards. The results, therefore, can be extrapolated to other centers with different camera systems using the same European guidelines. This study also has some limitations. The retrospective design of this study may introduce selection bias, which is inherent to cross-sectional studies. To minimize this bias, and although the sample size is somewhat limited, we included all patients who underwent an FDG PET/CT scan for FRI diagnostics during the inclusion period. After all, this study reflects clinical practice and represents one of the largest series investigating FDG PET/CT and lymph node assessment using both the consensus definition and standardized (EARL) reconstructions for diagnosing FRI.

## 5. Conclusions

The presence and number of locoregional lymph nodes with increased [18F]FDG-uptake alone has a poor diagnostic accuracy for FRI. The SUVmax of the ‘hottest’ lymph node showed moderate diagnostic performance. The addition of lymph node assessment to the regular 18F]FDG-PET/CT interpretation only marginally increases its diagnostic yield.

## Figures and Tables

**Table 1:** Patient characteristics.

Sex	
Male	88 (71%)
Female	36 (29%)
Age (mean + SD, years)	49 (SD 8.6)
Fracture location	
Humerus	4 (2.5%)
Ulna/radius	8 (6.5%)
Femur	36 (29%)
Tibia	69 (56%)
Foot	7 (6%)
Fracture type	
Open	71 (57%)
Closed	53 (43%)
Injury/scan interval (mean + SD, months)	79 (SD 11.7)
Last surgery/scan interval (mean + SD, months)	29 (SD 48.6)
Confirmed FRI	71 (51%)
Medical microbiology results	55 (77%)
Clinical follow up	16 (23%)

## Data Availability

The original contributions presented in this study are included in the article. Further inquiries can be directed to the corresponding author.
