# Correction: Bosch et al. Diagnostic Value of Increased [18F]FDG Uptake in Locoregional Lymph Nodes on PET/CT in Patients with Suspected Fracture-Related Infection. *Diagnostics* 2025, *15*, 616

**DOI:** 10.3390/diagnostics15151894

**Published:** 2025-07-29

**Authors:** Paul Bosch, Andor W. J. M. Glaudemans, Jean-Paul P. M. de Vries, Johannes H. van Snick, Justin V. C. Lemans, Janna van den Kieboom, Monique G. G. Hobbelink, Geertje A. M. Govaert, Frank F. A. IJpma

**Affiliations:** 1 Department of Surgery, University Medical Center Groningen, University of Groningen, 9712 CP Groningen, The Netherlands; paulbosch87@gmail.com (P.B.); j.p.p.m.de.vries@umcg.nl (J.-P.P.M.d.V.); f.f.a.ijpma@umcg.nl (F.F.A.I.); 2 Department of Nuclear Medicine and Molecular Imaging, University Medical Center Groningen, University of Groningen, 9712 CP Groningen, The Netherlands; j.h.van.snick@umcg.nl; 3 Department of Trauma Surgery, University Medical Center Utrecht, University of Utrecht, 3584 CS Utrecht, The Netherlands; j.v.c.lemans-3@umcutrecht.nl (J.V.C.L.); j.vandenkieboom-2@umcutrecht.nl (J.v.d.K.); g.a.m.govaert@umcutrecht.nl (G.A.M.G.); 4 Department of Radiology and Nuclear Medicine, University Medical Center Utrecht, University of Utrecht, 3584 CS Utrecht, The Netherlands; m.g.g.hobbelink@umcutrecht.nl

## 1. Error in Figure/Table

In the original publication [1], there was a mistake in Figure 1 as published. The numbers of FRI positive/FRI negative cases and positive/negative lymph nodes were switched in the figure. The corrected version of Figure 1 appears below. 

In the original publication, there was a mistake in Table 1 as published. The number of FRI positive cases was incorrect due to the aforementioned error in Figure 1. The corrected version of Table 1 appears below. The authors state that the scientific conclusions are unaffected. This correction was approved by the Academic Editor. The original publication has also been updated.

## 2. Text Correction

There was an error in the original publication. In the results section of the abstract, an incorrect number of confirmed FRI cases was mentioned. Section 3.2 contains incorrect numbers of confirmed FRI cases and their incorrect identification from Table 2. In Section 3.3, an error was made in transferring the number of FP, TP, FN and TN cases from the statistics tool to the manuscript. In the second paragraph of the discussion section, an error was made in the percentage of confirmed FRI patients with positive lymph nodes in our study. Corrections have been made to the Abstract, Sections 3.2. and 3.3, and the Discussion section.

*Abstract, Results Section*

In total, 124 patients were included in the analysis, with 53 cases of confirmed FRI. The presence of locoregional lymph nodes alone showed poor diagnostic accuracy (sensitivity, 55%; specificity, 68%; diagnostic accuracy, 62%). The number of active lymph nodes showed poor discriminative performance between FRI and non-infectious cases (AUC 0.63). Utilizing the SUVmax of the ‘hottest’ lymph nodes, moderate discriminative performance was revealed, with an AUC of 0.71. The optimal cutoff point (SUVmax 3.48) resulted in a sensitivity of 72%, a specificity of 78% and a diagnostic accuracy of 75%. A logistic regression model was fitted to calculate the added value of lymph node assessment to the regular [18F]FDG-PET/CT assessment. This resulted in a sensitivity of 71%, a specificity of 82% and a diagnostic accuracy of 76%.

*Section 3.2. FRI in Study Population*

Of the 124 included patients, 53 (43%) were diagnosed with FRI based on the AO/EBJIS consensus definition. Of the 53 FRI cases, 41 (77%) were diagnosed based on intra-operative deep tissue cultures, and 12 were diagnosed based on clinical confirmatory signs during a follow-up after at least 6 months after the first clinical suspicion of FRI.

*Section 3.3. Diagnostic Accuracy of Standard Assessment Protocol*

First, the diagnostic performance of the regular [18F]FDG-PET/CT assessment was calculated using crosstabs, yielding 39 true-positive results, 14 false-negative results, 55 true-negative results and 16 false-positive results. This resulted in a sensitivity of 74% (60–85%), a specificity of 77% (66–86%), and a diagnostic accuracy of 75% (67–83%).

*Section 4. Discussion, Paragraph 2*

Only one other study reported on the diagnostic value of locoregional lymph nodes in cases of suspected FRI [18]. Wang et al. reported good diagnostic accuracy for the SUVmax of inguinal lymph nodes in cases with suspected FRI. Their retrospective cohort study included 254 patients, with a definite diagnosis of FRI in 197 patients. The AO/EBJIS consensus definition was used as a reference standard for diagnosing FRI, though no information regarding follow-up was specified. They found a sensitivity of 86.8%, a specificity of 93.0% and an AUC of 0.939 for the SUVmax measurement in the ‘hottest’ inguinal lymph node. Furthermore, the diagnostic accuracy using the SUVmax in inguinal lymph nodes was higher than the diagnostic accuracy using the SUVmax at suspected FRI sites. Unfortunately, they did not report the number of patients with confirmed FRI that showed increased [18F]FDG uptake in locoregional lymph nodes. Furthermore, scan acquisition and quantitative analysis were not standardized. In our cohort, only 55% of patients who were diagnosed with FRI showed increased FDG uptake in locoregional lymph nodes.

The authors state that the scientific conclusions are unaffected. This correction was approved by the Academic Editor. The original publication has also been updated.

## Figures and Tables

**Figure 1:**
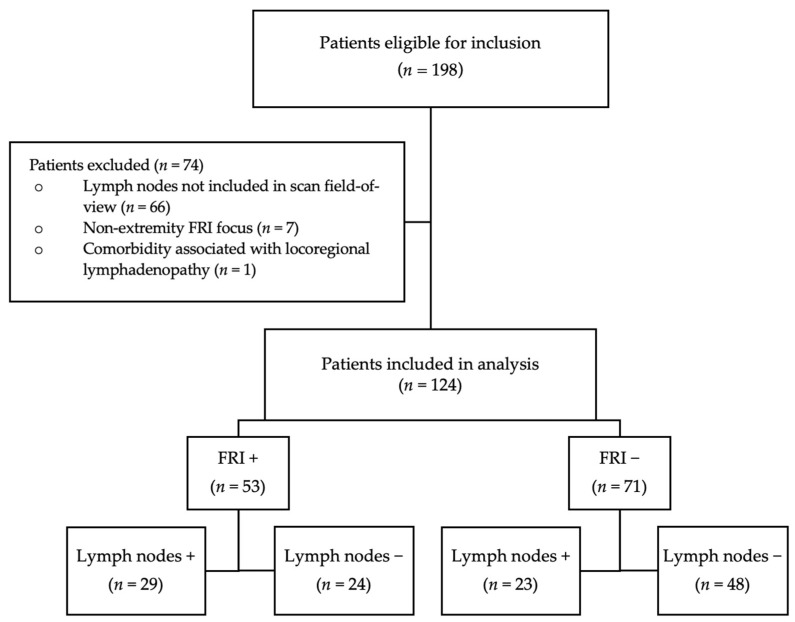
Flowchart of patient inclusion.

**Table 1:** Patient characteristics.

Sex	
Male	88 (71%)
Female	36 (29%)
Age (mean + SD, years)	49 (SD 8.6)
Fracture location	
Humerus	4 (2.5%)
Ulna/radius	8 (6.5%)
Femur	36 (29%)
Tibia	69 (56%)
Foot	7 (6%)
Fracture type	
Open	71 (57%)
Closed	53 (43%)
Injury/scan interval (mean + SD, months)	79 (SD 11.7)
Last surgery/scan interval (mean + SD, months)	29 (SD 48.6)
Confirmed FRI	53 (43%)
Medical microbiology results	41 (77%)
Clinical follow up	12 (23%)
